# Macrophage immunometabolic reprogramming impairs tissue regeneration in type 2 diabetes zebrafish model

**DOI:** 10.3389/fimmu.2025.1698674

**Published:** 2026-01-16

**Authors:** Leonel Witcoski Junior, Jordana Dinorá de Lima, André Guilherme Portela de Paula, Thais Sibioni Berti Bastos, Rebeca Bosso dos Santos Luz, Israel Henrique Bini, Matheus Brandemarte Severino, Luis Eduardo Alves Damasceno, Lais Cavalieri Paredes, Amanda Girardi Somensi, Elaine Cristina de Almeida Abreu, Lucas Brito de Souza Santos, Gabriel Costa Lourenço, Mariana Rodrigues Davanso, Rilton Alves de Freitas, Juliana Bello Baron Maurer, Niels Olsen Saraiva Câmara, José Carlos Alves Filho, Karin Braun Prado, Tárcio Teodoro Braga

**Affiliations:** 1 Department of Basic Pathology, Federal University of Paraná (UFPR), Curitiba, Paraná, Brazil; 2 Advanced Fluorescence Technologies Center, Federal University of Paraná (UFPR), Curitiba, Paraná, Brazil; 3 Department of Pharmacology, Ribeirão Preto Medical School, University of São Paulo (FMRP-USP), Ribeirão Preto, São Paulo, Brazil; 4 Department of Immunology, Institute of Biomedical Sciences, University of São Paulo (USP), São Paulo, Brazil; 5 Department of Genetics, Federal University of Paraná (UFPR), Curitiba, Paraná, Brazil; 6 Tuiuti University, Curitiba, Paraná, Brazil; 7 Department of Pharmacy, Federal University of Paraná, Curitiba, Paraná, Brazil; 8 Department of Biochemistry and Molecular Biology, Federal University of Paraná (UFPR), Curitiba, Paraná, Brazil

**Keywords:** immunometabolic reprogramming, macrophages, tissue regeneration, type 2 diabetes, zebrafish

## Abstract

**Introduction:**

Type 2 diabetes mellitus (T2D) is a metabolic disorder characterized by chronic hyperglycemia, insulin resistance, and meta-inflammation, which significantly compromise tissue regeneration. Although macrophage dysfunction is implicated in impaired wound healing in T2D, the immunometabolic mechanisms linking hyperglycemia to defective tissue repair remain incompletely understood.

**Methods:**

Zebrafish larvae were exposed to hyperglycemic conditions (4% dextrose) to establish a T2D-like model. Survival, glycemic and biochemical parameters were assessed, followed by caudal fin amputation to evaluate regenerative capacity. Total (Mpeg1^+^) and pro-inflammatory (Mpeg1^+^/TNF^+^) macrophages were quantified in vivo using confocal microscopy. Additionally, renal-derived macrophages were differentiated ex vivo under normoglycemic or hyperglycemic conditions and analyzed for mitochondrial function, reactive oxygen species (ROS) production, glucose uptake, and glycolytic metabolism using fluorescence probes and Seahorse assays.

**Results:**

Hyperglycemia induced severe metabolic dysregulation, including a 3.5-fold increase in lactate levels and elevated glycemia, and resulted in a 50% reduction in caudal fin regeneration at 72 hours post-injury. Hyperglycemic larvae exhibited a 2.3-fold increase in pro-inflammatory macrophages at the injury site. Ex vivo, macrophages exposed to hyperglycemic conditions showed a 64% reduction in mitochondrial mass, increased mitochondrial ROS production, enhanced glucose uptake, and elevated glycolytic activity, indicating a metabolic shift toward aerobic glycolysis.

**Discussion:**

These findings demonstrate that hyperglycemia drives immunometabolic reprogramming of macrophages, sustaining a pro-inflammatory phenotype that impairs tissue regeneration. The zebrafish T2D model provides a robust platform to investigate macrophage-driven immunometabolic mechanisms underlying defective wound healing and to explore therapeutic strategies targeting macrophage metabolism in T2D.

## 1 Introduction

Type 2 diabetes mellitus (T2D) represents a global public health emergency projected to reach 783 million people by 2045 (1). This multifactorial metabolic disorder arises from a complex interplay of genetic predisposition, environmental factors, and lifestyle behaviors, and is characterized by chronic hyperglycemia driven by insulin resistance and/or pancreatic β-cell dysfunction (2, 3). Additionally, metainflammation has emerged as a central mechanism in T2D pathogenesis, with macrophage polarization playing a key role (4, 5).

Under physiological conditions, these leukocytes maintain tissue homeostasis through a dynamic balance between proinflammatory and pro-resolving phenotypes (6, 7). In T2D, persistent hyperglycemia and insulin resistance favor the proinflammatory profile, increasing the production of TNF-α, IL-6 and reactive oxygen species (ROS), thereby perpetuating systemic inflammation and impairing tissue regeneration (2, 6).

The zebrafish (Danio rerio), sharing approximately 87% genetic homology with humans, has proven to be a powerful model for investigating the effects of hyperglycemia due to its optical transparency, high regenerative capacity, and genetic tractability (8, 9). However, critical gaps remain regarding (i) how hyperglycemia dynamically modulates macrophage polarization *in vivo*, and (ii) how hyperglycemia signaling influences regenerative processes (10).

Moreover, ex vivo derived macrophage represents a powerful model to investigate the role of hyperglycemia-induced metainflammation. Therefore, in addition to characterize macrophage phenotypes in hyperglycemic zebrafish larvae and assess their impact on tissue repair, the present study aims to analyze metabolic alterations in ex vivo-derived macrophages exposed to hyperglycemic conditions. We hypothesize that hyperglycemia induces a proinflammatory metabolic program in macrophages, thereby impairing regenerative outcomes.

## 2 Methods

### 2.1 Maintenance of zebrafish

Experimental animals were housed in the UFPR Animal Facility (Bioterio, Biological Sciences Sector, Polytechnic Center). Four days post-fertilization (dpf) zebrafish larvae were maintained in 25 mL of E3 solution (5 mM NaCl, 0.17 mM KCl, 0.33 mM CaCl_2_, 0.33 mM MgSO_4_; pH 7.2) containing methylene blue to inhibit fungal growth. Plates were incubated at 28.5 °C. All procedures in this study followed the Brazilian Directive for the Care and Use of Animals for Scientific and Teaching Purposes (DBCA) established by the National Council for the Control of Animal Experimentation (CONCEA), as well as international guidelines for animal experimentation. This project was approved by the Animal Use Ethics Committee (CEUA) of the Federal University of Paraná (UFPR; protocol 23075.029356/2024-3).

### 2.2 Type 2 diabetes model

To assess larval survival under hyperglycemic conditions, we performed Kaplan–Meier survival analysis. Twenty-five larvae per experimental group were maintained in 25 mL of solution and exposed from 4 dpf to glucose concentrations of 3.5%, 4% and 4.5% for seven days, following Singh et al. (11) “High glucose levels affect retinal patterning during zebrafish embryogenesis.” Mortality was recorded daily, and medium was renewed every 24 h to prevent microbial growth.

For the definitive model of T2D, 4 dpf larvae were immersed in 25 mL of 4% dextrose (Mono, Extrapura – Titan) dissolved in E3 with methylene blue. Medium was changed daily to avoid fungal or microbial contamination. Control larvae remained in E3 with methylene blue. Survival curves were generated by Kaplan–Meier analysis and compared by log-rank (Mantel–Cox) test to identify statistically significant differences among groups.

### 2.3 Measurement of glycemia, total protein and lactate

Groups of 25 larvae exposed from 4 dpf to glucose (4%) and control (normoglycemic) for seven days were anesthetized in 20 mL tricaine (164 mg/L) for five minutes and rinsed three times with distilled water. Larvae were then transferred to 1.5 mL microtubes and homogenized in 200 µL Milli-Q water using a vortex (three minutes, maximum speed). Samples were centrifuged at 12,000 rpm for five minutes, and the supernatant collected for analysis.

Glycemia was quantified with the Liquiform Glucose kit (GOD-Trinder; Ref. 133, MS 10009010236) according to manufacturer instructions. Total protein concentration was measured by the Biuret method using Labtest kit (Ref. 99, MS 1000910080). Lactate was assessed with the Labtest Enzymatic Lactate kit (Trinder method; Ref. 138, MS 10009010258) following the manufacturer’s protocol.

### 2.4 Quantification of total and proinflammatory macrophages

The phenotypic profile of total and proinflammatory macrophages in zebrafish larvae under diabetic and control conditions was assessed using the transgenic line Tg(Mpeg1:mCherry/TNFα:GFP). In this line, the macrophage-specific mpeg1 promoter drives expression of the red fluorescent protein mCherry (ex:587 nm/em:610 nm), labeling all macrophages in red. The green fluorescent protein GFP (ex:470 nm/em:509 nm) is placed under control of the tnfa promoter, indicating TNFα expression.

Inflammation was induced by transecting the caudal fin just dorsal to the notochord in glucose- and control- exposed larvae over a 7-day exposure period. Larvae were imaged at 24 and 472 hours post-injury (hpi) on a Nikon A1R MP+ confocal microscope at the Centro de Tecnologias Avançadas em Fluorescência (CTAF), at Biological Sciences building, at UFPR. Images were acquired at one frame every 4 seconds, 1024×1024 resolution, with 2.5 µm z-steps over a 55 µm range. All data were saved as ND2 files and analyzed in ImageJ (Fiji).

### 2.5 Analysis of regenerated area

Regeneration of the caudal fin was compared between hyperglycemic and control larvae over a 7-day exposure period. On day 7, larvae were anesthetized with tricaine (164 mg/L) for 5 minutes, then the caudal fin was amputated vertically at the notochord under a stereomicroscope. Regeneration was imaged by confocal microscopy at 24 and 72 hpi. Regenerated area was quantified in ImageJ by measuring the newly formed tissue and compared across treatment groups.

### 2.6 Zoledronic encapsulation by acid-loaded liposomes

Liposomes were prepared by dissolving 1,2-dioleoyl-sn-glycero-3-phosphoethanolamine (DOPE, 2.5 mM) and distearoylphosphatidylcholine (DSPC, 248.6 µM) in chloroform (12). The solvent was removed by rotary evaporation at 50 °C, forming a lipid film. Hydration was carried out in a glycerin bath (70 °C, 1 h, continuous agitation) with either zoledronic acid (400 µg/mL) or 1× PBS (control). Liposome size and zeta potential were determined by dynamic light scattering using a Malvern Zeta Nano Series instrument.

Normoglycemic zebrafish larvae (9 dpf) were anesthetized with tricaine (164 mg/L, 3–5 min) and positioned laterally on 1% agar/E3 medium. Microinjection into the yolk sac was performed with borosilicate micropipettes (tip diameter ≈ 20–30 µm) at a volume of 300 nL per larva. Experimental groups received zoledronic-acid liposomes, whereas control groups received PBS-loaded liposomes. Post-injection, larvae were maintained at 28.5 °C in E3 medium containing methylene blue, with viability monitored every 12 h.

### 2.7 Ex vivo macrophage obtainment

Ex vivo culture of renal-derived macrophages was performed using adult Tg(Mpeg1:mCherry) fish. Adults were euthanized on ice for 5 minutes, and kidneys were dissected into ice-cold PBS 1× (pH 7.4) containing antibiotics to prevent contamination: tetracycline (1 µg/mL), penicillin-streptomycin (1%), gentamicin (10 µg/mL), chloramphenicol (15 µg/mL), ampicillin (100 µg/mL), and amphotericin B (250 ng/mL). Kidneys were passed through a 40 µm cell strainer, centrifuged at 300 g for 5 minutes, and the cell pellet resuspended in Leibovitz L-15 medium (Gibco L1518) supplemented with 20% heat‐inactivated FBS and the same antibiotic mix. Medium was conditioned with 30% L929 cell-culture supernatant to stimulate macrophages. Cells were plated in 24-well plates and medium refreshed on days 1, 2, and 5. Cultures were maintained at 28 °C in 5% CO_2_.

To evaluate the metabolic impact of hyperglycemia on renal macrophages, cultures were maintained under two distinct experimental conditions: normoglycemic condition - L-15 medium supplemented with dextrose at 3.466 mM concentration (3.123 mg in 5 mL of medium), corresponding to physiological glycemic levels in zebrafish; and hyperglycemic condition - L-15 medium supplemented with dextrose at 4% increased concentration relative to normoglycemic condition (3.248 mg in 5 mL of medium, equivalent to 3.605 mM), reproducing the hyperglycemic conditions used in *in vivo* experiments. Specific dextrose concentrations were maintained throughout the culture period through medium renewal on days 1, 2, and 5, ensuring consistent exposure to respective glycemic conditions over the 7-day culture period.

### 2.8 Mitochondrial probe quantification

After 7 days of treatment, ex vivo macrophages were stained to assess mitochondrial reactive oxygen species (ROS) and mass by flow cytometry. Cells were incubated with 5 µM MitoSOX™ Red (Invitrogen) for 30 minutes at 28 °C to detect mitochondrial superoxide, then with 250 nM MitoTracker™ Red (Invitrogen) for 30 minutes at 28 °C in the dark to assess mitochondrial mass. Cells were washed in PBS 1× and chilled on ice for 5 minutes to detach. Data were acquired on a BD FACSCanto™ cytometer and analyzed in FlowJo^®^ v10.7 to compare fluorescence intensity between normoglycemic and hyperglycemic groups. Macrophages were also imaged at 24 and 472 hours post-injury (hpi) on a Nikon A1R MP+ confocal microscope.

### 2.9 Metabolic analysis using seahorse

To assess cellular glycolytic dependence, we employed the Seahorse XFe24 Analyzer (Agilent). On Day 1, 4×10^4 cells were seeded per well of an XF24 plate in 150 µL of Seahorse XF Base Medium DMEM supplemented with 20 mM glucose, 2 mM L-glutamine, and 1 mM sodium pyruvate. The sensor cartridge was hydrated in 1 mL of Seahorse XF calibrant (pH 7.4) at 28 °C for 24–72 h in a CO_2_-free environment. On Day 2, assay medium in each well was replaced with 500 µL of pre-warmed base medium (28 °C), and sequential injections were performed as follows: 25 µL of 1 M glucose into Port A (final concentration 10 mM, total volume 175 µL); 25 µL of 1 mg/mL oligomycin into Port B (final concentration 1 µg/mL, total volume 200 µL); and 25 µL of 200 mM 2-deoxy-D-glucose into Port C (final concentration 22 mM, total volume 225 µL). Extracellular acidification rate (ECAR) was recorded in real time after each addition. For normalization, cells were stained with Hoechst 33342 and imaged on a Cytation 1/5 reader (DAPI filter). Automated nuclear counts were obtained using Agilent Seahorse XF Imaging and Cell Counting software, and ECAR values were adjusted to cell number.

### 2.10 Statistical analysis

Statistical analyses were performed using GraphPad Prism 10.0 (GraphPad Software, San Diego, CA, USA). Data are presented as mean ± standard error of the mean (SEM) from at least three independent biological replicates unless otherwise stated. Data normality was assessed using the Shapiro-Wilk test. For comparisons between two groups, unpaired two-tailed Student’s t-tests were applied when data showed normal distribution, or Mann-Whitney U tests were used for non-parametric data. For multiple group comparisons, one-way analysis of variance (ANOVA) followed by Tukey’s multiple comparisons *post hoc* test was performed for normally distributed data, or Kruskal-Wallis test followed by Dunn’s multiple comparisons test was used for non-parametric data. Two-way ANOVA followed by Sidak’s multiple comparisons test was applied when analyzing the interaction between two factors (e.g., treatment and time). Statistical significance was set at p < 0.05, with specific p-values reported when p < 0.001.

## 3 Results

### 3.1 Hyperglycemia impaired metabolic homeostasis in zebrafish larvae indicate effective T2DM mimicry

**Figure 1:**
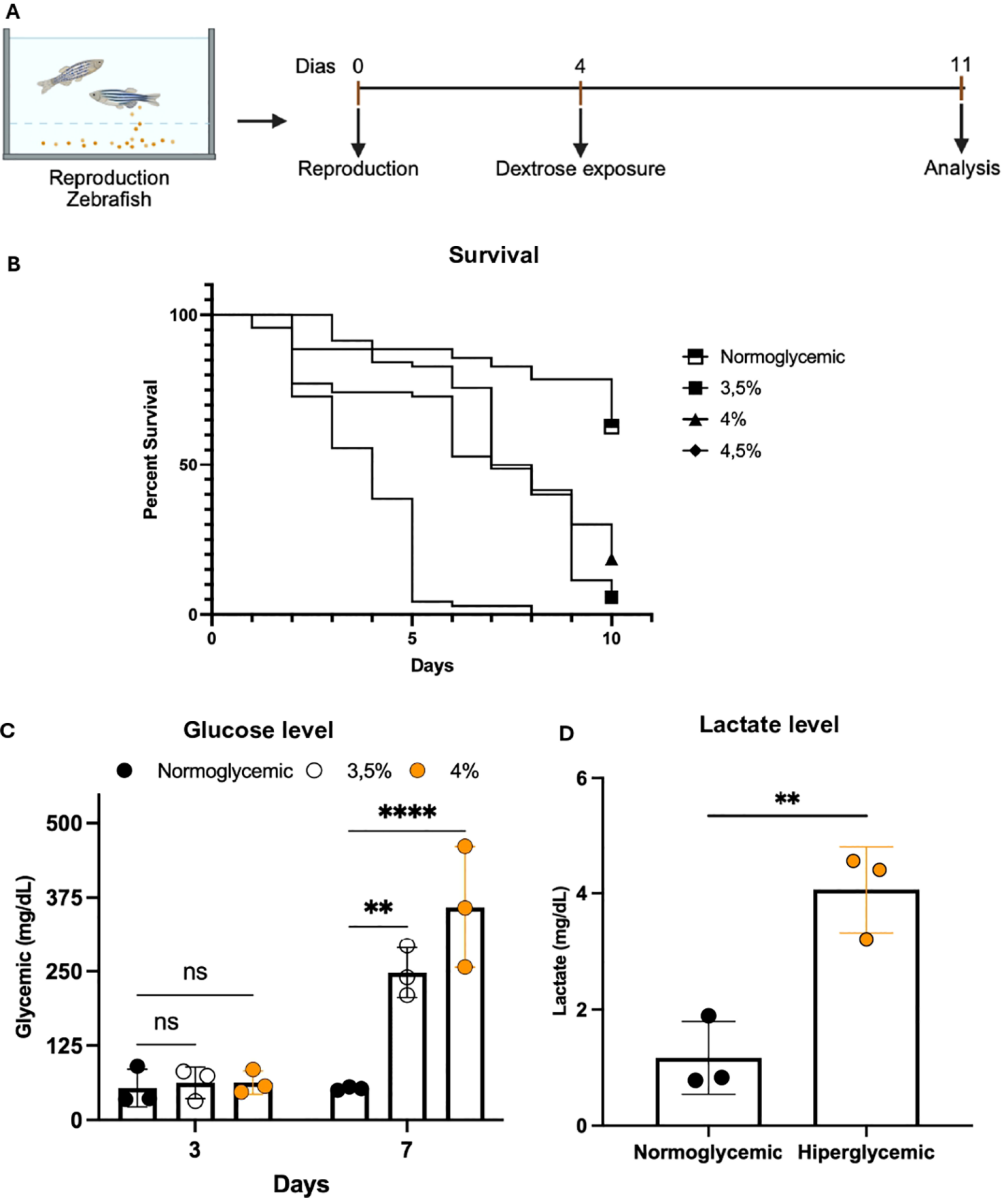
Impact of dextrose-induced hyperglycemia on zebrafish larvae survival, metabolism and insulin-signaling proteins. **(A)** Experimental design. Reproductive adult zebrafish were maintained under normoglycemic conditions until spawning (day 0), then larvae were exposed from 4 days post-fertilization (dpf) to either control medium or medium containing 3.5%, 4% or 4.5% dextrose. Endpoints were assessed on day 11 (14 dpf). **(B)** Kaplan–Meier survival curves for larvae exposed to normoglycemic (blue) or hyperglycemic conditions (3.5% red, 4% green, 4.5% purple dextrose). Shaded areas denote 95% confidence intervals. A log-rank test indicated significant differences among groups (χ² = 191.0, df = 3, p < 0.0001) with a dose-dependent mortality trend (log-rank trend test: χ² = 119.1, df = 1, p < 0.0001). n = 70 larvae per group per experiment; three independent experiments. **(C)** Whole-body glucose concentrations (mg/dL) at 3 and 7 dpe in normoglycemic (black circles), 3.5% (gray diamonds) and 4% (light gray squares) dextrose groups. At 3 dpe, no significant differences were detected (two-way ANOVA: Treatment F(2,12) = 16, p = 0.0004; Time F(1,12) = 48, p < 0.0001; Interaction F(2,12) = 14, p = 0.0007; Tukey’s *post-hoc* ns, p > 0.05). At 7 dpe, both hyperglycemic groups displayed higher glucose than control (3.5%: **p < 0.01; 4%: ****p < 0.0001), and the 4% group exceeded the 3.5% group (*p < 0.05). Mean ± SEM, n = 25 larvae per group per experiment. **(D)** Whole-body lactate levels (mg/dL) at 7 dpe in control (gray bar) and hyperglycemic (4% dextrose, light gray bar) larvae. We observed elevated lactate in hyperglycemic larvae (4.07 ± 0.56 vs. 1.17 ± SEM mg/dL; **p = 0.0068; unpaired two-tailed t-test with Welch’s correction: t = 5.139, df = 4). Data are mean ± SEM, n = 25 larvae per group per experiment (total n = 75).

Zebrafish larvae at 4 days post-fertilization (dpf) were exposed to increasing dextrose concentrations (3.5%, 4%, and 4.5%) for 7 days, with daily mortality monitoring (**Figure 1A**). Metabolic parameters (glycemia, lactate) were analyzed on the 7th day post-dextrose exposure (dpe). Exposure to dextrose concentrations above 4.5% resulted in 100% mortality at 5 dpe (**Figure 1B**). At 4% concentration, we observed 50% cumulative mortality after 7 days, while 3.5% concentration did not cause significant mortality, indicating dose-dependent toxicity (**Figure 1B**).

While larvae exposure to normoglycemic condition (E3 medium without dextrose) maintained stable glycemia at 70 ± 10 mg/dL at 3 and 7 dpe, larvae exposed to 3.5% dextrose presented glycemic levels of 200 ± 40 mg/dL, and larvae exposed to 4% dextrose exhibited baseline plasma glucose levels of 350 ± 40 mg/dL at 7 dpe (**Figure 1C**). Moreover, blood lactate concentration increased 3.5-fold in the hyperglycemic group (4%) compared to control (12.0 ± 1.8 mM vs. 3.4 ± 0.5 mM), reflecting metabolic shift toward anaerobic glycolysis (**Figure 1D**).

Therefore, these results indicate that exposure to increasing dextrose concentrations in zebrafish larvae leads to a dose-dependent metabolic response characterized by severe hyperglycemia and increased blood lactate, evidencing metabolic shift toward anaerobic glycolysis. These data highlight the toxic impact of acute elevated dextrose concentrations on larval metabolism and survival, establishing an effective T2DM model for subsequent macrophage immunometabolic studies.

### 3.2 Hyperglycemia compromises tissue regeneration and increases inflammatory macrophages in zebrafish larvae

**Figure 2:**
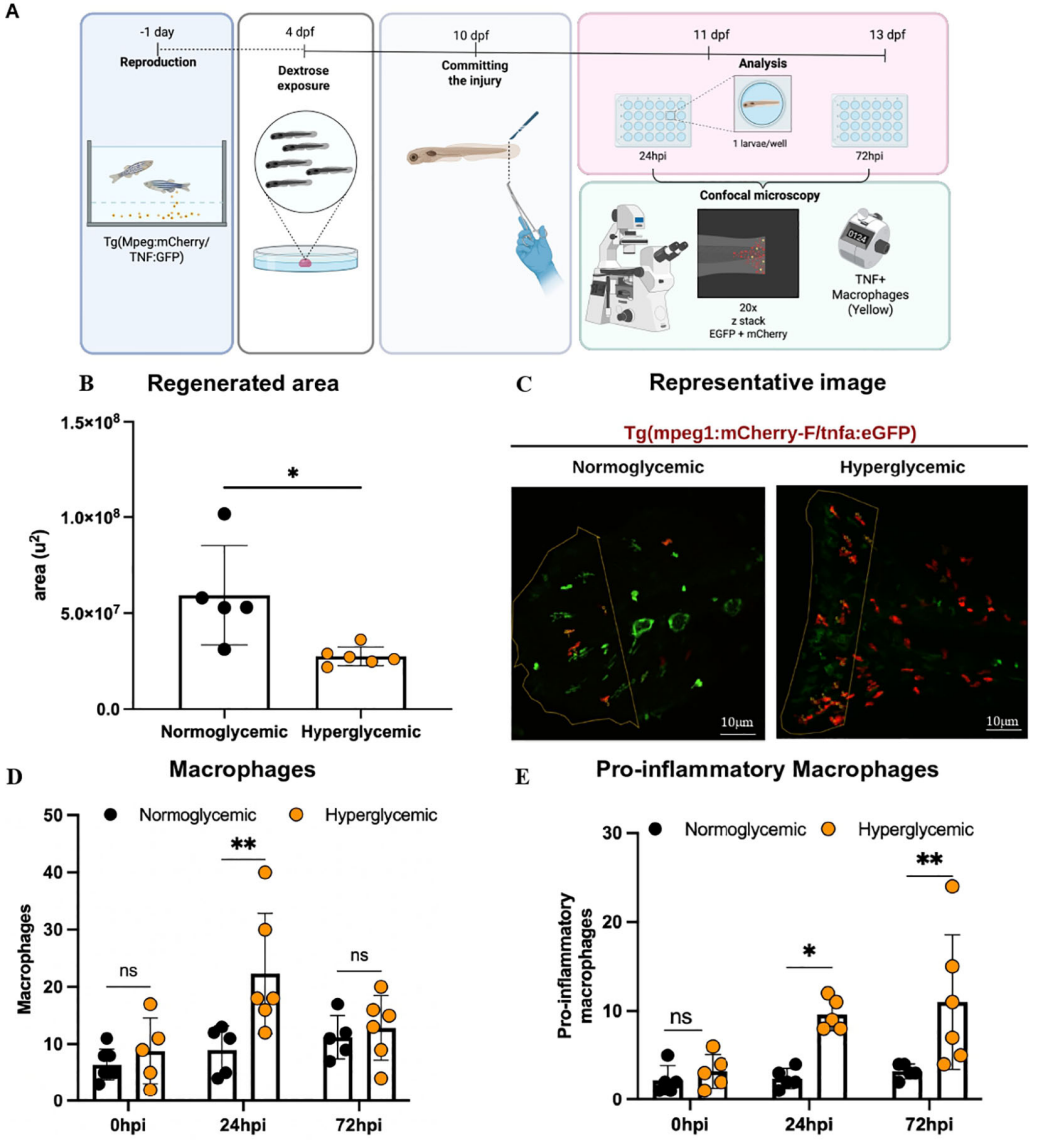
Hyperglycemia impairs tissue regeneration and promotes inflammatory macrophage accumulation in zebrafish larvae. **(A)** Experimental timeline for caudal fin amputation and analysis in transgenic Tg(mpeg:mCherry/TNF: GFP) zebrafish larvae. Larvae were exposed to normoglycemic (control) or hyperglycemic (4% dextrose) conditions from 4 days post-fertilization, followed by caudal fin amputation at 10 dpf. Macrophage quantification and regeneration analysis were performed at 0, 24, and 72 hours post-injury (hpi). Confocal microscopy was used to visualize macrophages (red, mCherry) and pro-inflammatory macrophages (yellow, TNF+). **(B)** Temporal progression of regenerated area quantification. Line graphs illustrate the kinetics of total macrophages (left) and pro-inflammatory macrophages (right) from 0 to 72 hpi. The arrow indicates the time of injury. Error bars represent SEM. Statistical analysis by two-way ANOVA revealed significant effects of both group and time for both outcomes, with no significant interaction. Caudal fin regeneration was significantly impaired in hyperglycemic larvae at 72 hpi (2.75×10^7^ ± 1.0×10^6^ μm²) compared to normoglycemic controls (5.93×10^7^ ± 1.0×10^6^ μm²; *p = 0.0153, η² = 0.498; unpaired t-test). **(C)** Representative confocal microscopy images of the injury site showing macrophage infiltration in normoglycemic and hyperglycemic larvae at 72 hpi. Green fluorescence indicates tissue autofluorescence, red shows total macrophages (mCherry), and yellow represents pro-inflammatory macrophages (TNF+). Scale bar represents specified magnification. **(D)** Total macrophage quantification at the injury site. Bar graphs show macrophage counts in normoglycemic (black bars) and hyperglycemic (gray bars) larvae at 0, 24, and 72 hpi. Individual data points represent single animals. Hyperglycemic larvae exhibited significantly higher total macrophage numbers at 24 hpi (**p = 0.0032). Data are mean ± SD, n = 10–11 animals per group/time point. **(E)** Pro-inflammatory macrophage quantification at the injury site. Pro-inflammatory macrophages (TNF+ cells) were significantly elevated in hyperglycemic larvae at both 24 hpi (*p = 0.0135) and 72 hpi (**p = 0.0049) compared to normoglycemic controls. Data are mean ± SD, n = 10–11 animals per group/time point.

To evaluate tissue regeneration and the inflammatory process in hyperglycemic conditions, transgenic zebrafish larvae Tg(mpeg:mCherry/TNF: GFP) were exposed to 4% dextrose for 7 days and then subjected to caudal fin amputation (**Figure 2A**). Temporal analysis of regeneration showed that at 24 hours post injury (hpi), the regenerated area showed no significant difference between groups (approximately 5×10^5^ µm² in normoglycemic and 4×10^5^ µm² in the hyperglycemic group) (**Figure 2B**). However, hyperglycemia induced a significant reduction in the regenerative capacity of zebrafish at 72 hpi - the regenerated area in the hyperglycemic group was 50% smaller (5 × 10^7^ µm²) compared to the normoglycemic group (10 × 10^7^ µm²; p < 0.05) (**Figure 2D**).

Due to its importance in the regenerative process, we next evaluated macrophage number at the injury site (4). Although the total number of macrophages did not differ between normoglycemic and hyperglycemic groups before injury at 6 dpe, total macrophage numbers are higher in hyperglycemic group at 24 hpi when compared to normoglycemic group (**Figure 2D**). However, such difference is lost at 72 hpi, as shown by quantitative analysis (**Figure 2D**) and representative confocal microscopy images (**Figure 2C**). On the other hand, phenotypic analysis revealed a 2.5-fold increase in the proportion of inflammatory macrophages (marked by Mpeg/TNF) in the hyperglycemic group at both 24 and 72 hpi (p < 0.01; **Figure 2E**, **Supplementary File 2**). These results suggest that hyperglycemia directly interferes with the regenerative process, altering the inflammatory profile at the injury site. Hyperglycemia not only compromised caudal fin regeneration in zebrafish but also altered the local inflammatory profile, suggesting that increased inflammatory macrophages predicts delayed tissue regeneration under hyperglycemic conditions.

### 3.3 Macrophage depletion impairs tissue regeneration in zebrafish larvae

**Figure 3:**
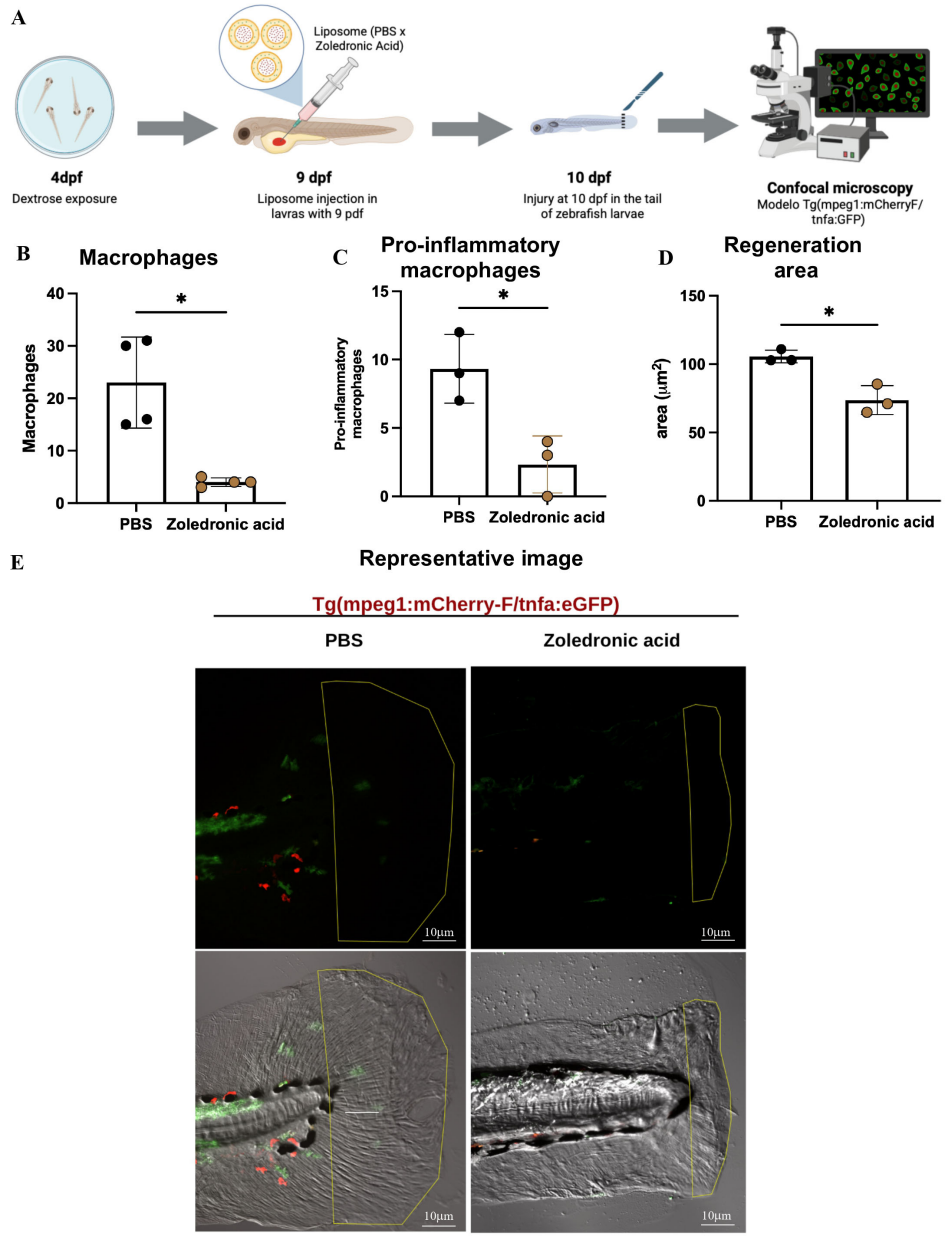
Macrophage depletion impairs tissue regeneration in zebrafish larvae. **(A)** Experimental timeline showing liposome injection at 9 dpf for macrophage depletion, tail amputation at 10 dpf, and confocal microscopy analysis using Tg(mpeg1:mCherry-F/tnfa:eGFP) transgenic line. **(B)** Quantification of total macrophages shows significant reduction in zoledronic acid-treated larvae compared to SHAM controls (mean difference = -19.00 ± 4.359, P = 0.0215, n = 4 per group). **(C)** Pro-inflammatory macrophage counts demonstrate marked decrease following zoledronic acid treatment (mean difference = -7.000 ± 1.886, P = 0.0219, n = 3 per group). **(D)** Regeneration area measurements reveal impaired tissue repair in macrophage-depleted larvae (mean difference = -31.81 ± 6.658 μm², P = 0.0214, n = 3 per group). **(E)** Representative confocal microscopy images of tail regeneration in SHAM (left) and zoledronic acid-treated (right) larvae at 24 hpa. Green fluorescence indicates macrophages (mpeg1:mCherry), red fluorescence shows pro-inflammatory markers (tnfa:eGFP). Yellow outline delineates regeneration area. Scale bars = 10 μm. Data are presented as mean ± SEM. Statistical significance was determined using unpaired t-test with Welch’s correction. *P < 0.05 was considered statistically significant. dpf, days post-fertilization; hpa, hours post-amputation.

To further assess the role of macrophages in the regenerative process, we injected zoledronic acid-loaded liposomes into 6 dpe larvae, and 24 h later we amputated the caudal fin of hyper- and normoglycemic animals (**Figure 3A**). Notably, all hyperglycemic larvae treated with zoledronic acid-loaded liposomes died within 24 hours post-injection, demonstrating that macrophages are essential for survival under hyperglycemic conditions (data not shown). This 100% mortality rate in the hyperglycemic + macrophage depletion group contrasted sharply with the survival of normoglycemic animals subjected to the same treatment, highlighting the critical dependency on macrophages during metabolic stress.

Due to the complete mortality of hyperglycemic animals following macrophage depletion, subsequent analyses of regenerative capacity and macrophage quantification were performed exclusively in the normoglycemic group. In these animals, zoledronic acid treatment resulted in significant macrophage reduction compared to PBS-loaded liposome controls (p = 0.0215; n = 4) (**Figure 3B**). Pro-inflammatory macrophages specifically decreased by 7.00 ± 1.89 cells (p = 0.0219; n = 3) (**Figure 3C**). Moreover, regenerated fin area declined by 31.81 ± 6.66 μm² in macrophage-depleted larvae compared with PBS-loaded liposome-injected animals (p = 0.0214; n = 3), corresponding to ~30% loss of regenerative capacity (**Figures 3D, E**).

Altogether, these data demonstrate the critical role of macrophages not only in physiological processes such as fin fold regeneration, but also in survival under hyperglycemic stress. The lethal outcome of macrophage depletion in hyperglycemic conditions strongly supports our hypothesis that macrophage functional alterations - such as those induced by hyperglycemia - underlie the regenerative deficits observed in T2D, while simultaneously revealing their essential role in maintaining homeostasis during metabolic dysfunction.

### 3.4 Ex vivo macrophages from hyperglycemic animals display a glycolytic profile with mitochondrial dysfunction

**Figure 4:**
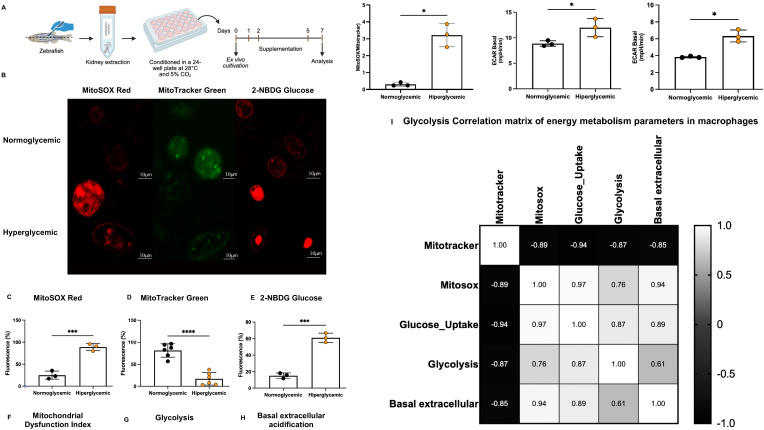
Hyperglycemia induces metabolic reprogramming in ex vivo macrophages characterized by mitochondrial dysfunction and glycolytic shift. **(A)** Experimental workflow for ex vivo macrophage isolation and conditioning. Zebrafish kidney cells were extracted, conditioned in 24-well plates at 28 °C with 5% CO_2_, and cultured for 7 days under normoglycemic or hyperglycemic conditions. Immunometabolic analysis was performed using flow cytometry and confocal microscopy with specific fluorescent probes. **(B)** Representative confocal microscopy images of ex vivo macrophages under normoglycemic and hyperglycemic conditions. Images show MitoSOX Red (mitochondrial ROS, red fluorescence), MitoTracker Green (mitochondrial mass, green fluorescence), and 2-NBDG (glucose uptake, red fluorescence). Scale bar = 10 μm. Hyperglycemic macrophages exhibit reduced mitochondrial mass, increased ROS production, and enhanced glucose uptake compared to normoglycemic controls. **(C)** Quantification of mitochondrial ROS production by MitoSOX Red fluorescence intensity. Hyperglycemic macrophages demonstrated significantly elevated ROS generation (88.67 ± 4.81%) compared to normoglycemic controls (25.20 ± 9.09%; ****p < 0.0001, unpaired t-test with Welch’s correction). **(D)** Quantification of mitochondrial mass by MitoTracker Green fluorescence intensity. Hyperglycemic macrophages showed significant reduction in mitochondrial mass (16.98 ± 2.47%) relative to normoglycemic controls (81.43 ± 3.11%; ****p < 0.0001, unpaired t-test with Welch’s correction). **(E)** Quantification of glucose uptake by 2-NBDG fluorescence intensity. Hyperglycemic macrophages exhibited significantly higher glucose uptake (61.20 ± 3.52%) compared to normoglycemic controls (15.20 ± 1.53%; ***p < 0.001, unpaired t-test with Welch’s correction). **(F)** Mitochondrial dysfunction index calculated as MitoSOX/MitoTracker ratio. Hyperglycemic macrophages presented significantly increased dysfunction index (3.20 ± 0.32) relative to normoglycemic controls (0.31 ± 0.04; *p < 0.05, unpaired t-test with Welch’s correction). **(G)** Glycolytic activity measured by basal extracellular acidification rate (ECAR). Hyperglycemic macrophages showed significantly higher glycolytic ECAR (11.98 ± 1.075 mpH/min) compared to normoglycemic controls (8.868 ± 1.075 mpH/min; *p = 0.0442, unpaired t-test). **(H)** Basal extracellular acidification rate related to basal respiration. Hyperglycemic macrophages demonstrated elevated ECAR (6.333 ± 0.4146 mpH/min) relative to normoglycemic controls (3.833 ± 0.4146 mpH/min; *p = 0.0246, Welch’s t-test). **(I)** Correlation matrix of energy metabolism parameters showing Pearson correlation coefficients (r) between mitochondrial mass (Mitotracker), mitochondrial ROS (Mitosox), glucose uptake, glycolysis, and basal extracellular acidification. Color scale ranges from blue (positive correlation, r → +1.0) to orange (negative correlation, r → -1.0). Strong negative correlations exist between mitochondrial mass and all other parameters (r = -0.853 to -0.937), with very strong positive correlation between mitochondrial ROS and glucose uptake (r = 0.971, p = 0.001).

Given that macrophages were being metabolically and functionally impacted in inflammatory contexts after dextrose exposure, we performed immunometabolic analyses of renal marrow-derived macrophages from zebrafish under hyper- and normoglycemic conditions. It is noteworthy that cells were kept under hyper- and normoglycemic media ex vivo, following the differentiation and conditioning protocol (**Figure 4A**).

Mitochondrial mass of cells exposed to hyperglycemic conditions was reduced (p < 0.01), showing an average decrease of 64.45% compared to the normoglycemic group (**Figures 4B, D**). Additionally, these cells demonstrated increased mitochondrial ROS production (p < 0.01), with an average difference of 63.6% between groups, as evidenced by quantification of MitoSOX Red probe fluorescence (**Figure 4C**). Furthermore, cells conditioned in the hyperglycemic environment were able to internalize a greater amount of 2-NBDG, suggesting increased utilization of the glycolytic pathway compared to macrophages maintained in normoglycemic medium (p < 0.01) (**Figure 4E**).

The mitochondrial dysfunction index, calculated as MitoSOX/MitoTracker ratio, demonstrated significant mitochondrial function impairment in the hyperglycemic group (**Figure 4F**). Results from the Extracellular Acidification Rate (ECAR) assay demonstrated an increase in both basal extracellular acidification rate (**Figure 4G**) and glucose consumption (**Figure 4H**) in the hyperglycemic group compared to the normoglycemic one (p < 0.05).

The correlation matrix of energy metabolism parameters revealed robust relationships between different metabolic markers, highlighting strong negative correlations between mitochondrial mass and all other parameters (r = -0.853 to -0.937), as well as very strong positive correlation between mitochondrial ROS and glucose uptake (r = 0.971, p = 0.001) (**Figure 4I**).

These findings demonstrate great glucose consumption of macrophages and glycolysis as their primary energy source when maintained in a hyperglycemic condition. Hyperglycemic environment reprograms macrophage metabolism, promoting an adaptation that favors aerobic glycolysis as their predominant metabolic strategy.

## 4 Discussion

The present study demonstrates that T2DM compromises tissue regeneration and alters the inflammatory profile in zebrafish larvae, corroborating clinical observations in humans and expanding our understanding of the mechanisms underlying this condition (2). The reduction in regenerated area in the hyperglycemic group reflects impaired wound healing, already well-documented in studies with people with T2DM (13). This significant delay in tissue repair is intimately linked to the metabolic and inflammatory alterations observed. The increase in the proportion of proinflammatory macrophages (mpeg1+/TNF+ cells) in the hyperglycemic group at 72 hpi is consistent with human studies reporting an accumulation of proinflammatory macrophages in diabetic wounds (6). This persistence of inflammatory macrophages contributes to a chronic proinflammatory environment, impairing the transition to later phases of tissue repair and remodeling (14).

Our hyperglycemic zebrafish model offers unique advantages for studying immunometabolic mechanisms involved in T2DM, allowing *in vivo* visualization of inflammatory and regenerative processes (8). The ability to simultaneously quantify regenerated area and specific proinflammatory macrophages overcomes limitations of previous studies, providing direct evidence of the relationship between hyperglycemia, meta-inflammation, and impaired tissue regeneration. The establishment of sustained hyperglycemia with accompanying lactate elevation demonstrates effective metabolic dysregulation characteristic of T2DM pathophysiology (15).

Furthermore, our results on metabolic reprogramming of ex vivo macrophages under hyperglycemic conditions, including reduced mitochondrial mass and increased ROS production, align with recent studies in mammalian T2DM models (16–18). This shift toward a glycolytic profile in macrophages exposed to hyperglycemia may explain their persistence in the pro-inflammatory phenotype, contributing to delayed wound healing (19, 20). The correlation matrix revealing strong relationships between mitochondrial dysfunction parameters and glycolytic markers provides mechanistic insight into how hyperglycemia drives immunometabolic reprogramming in macrophages (21).

In conclusion, our findings establish a mechanistic link between T2DM-induced metabolic alterations and macrophage-mediated impairment of tissue regeneration in zebrafish (11, 22). This model offers a robust platform for future investigations into therapeutic interventions aimed at modulating the metabolic and inflammatory profile of macrophages in the context of T2DM (23). Such approaches have the potential to significantly improve wound healing outcomes in diabetic patients, representing an important advancement in the field of regenerative medicine and treatment of diabetes-associated chronic wounds, which greatly impair the quality of life and life expectancy of patients with this pathophysiology (24, 25).

## Data Availability

The original contributions presented in the study are included in the article/supplementary material. Further inquiries can be directed to the corresponding authors.
