# Supplementary figures and images for: Macrophage immunometabolic reprogramming impairs tissue regeneration in type 2 diabetes zebrafish model
Source: Front Immunol. 2026 Jan 16;16:1698674. doi: 10.3389/fimmu.2025.1698674 (PMC12856266; doi:10.3389/fimmu.2025.1698674)

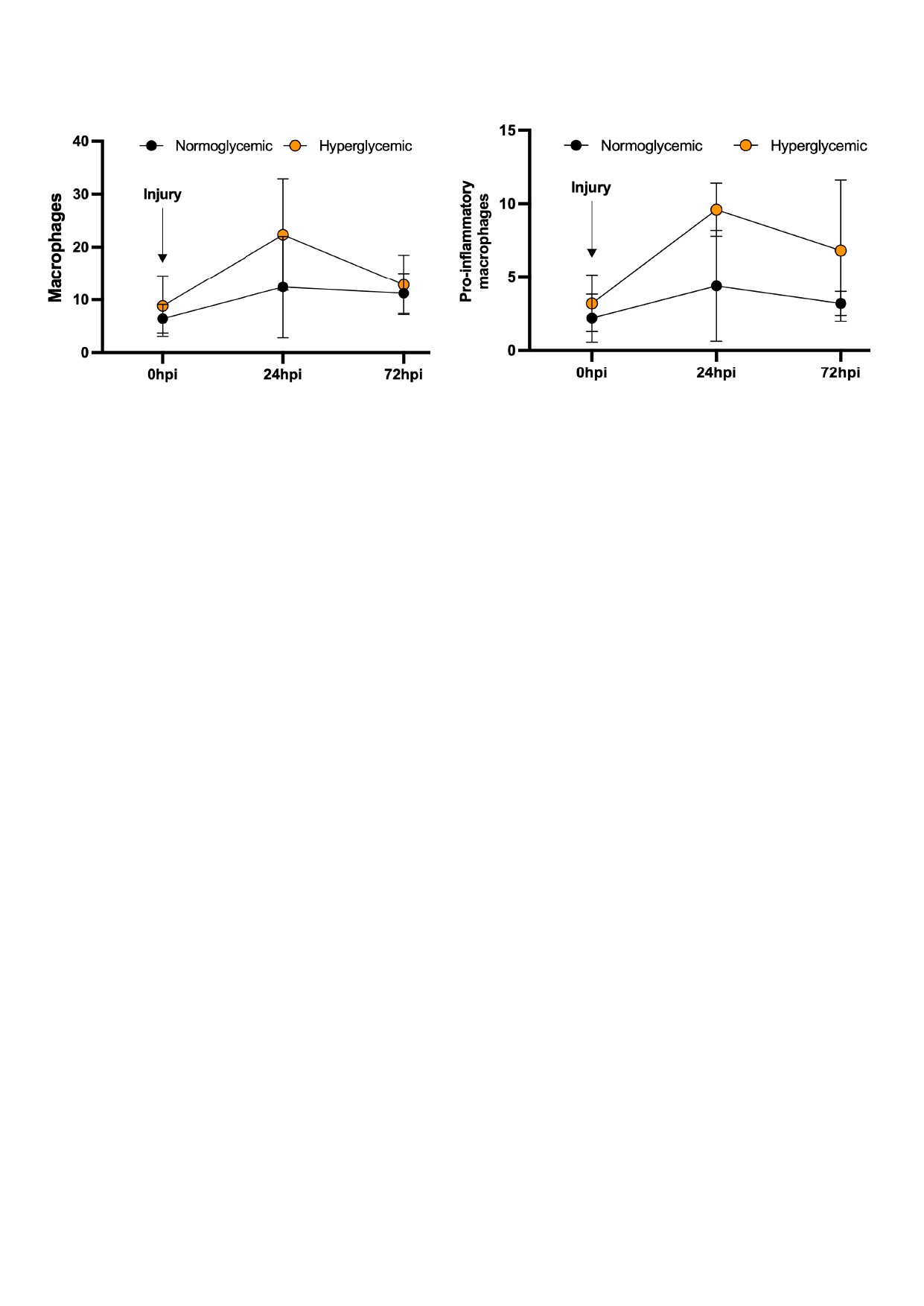

Supplement: Supplementary file 1 [file Supplementaryfile2.jpg]
